# Use of Brassica Plants in the Phytoremediation and Biofumigation Processes

**DOI:** 10.3390/ijms12117760

**Published:** 2011-11-09

**Authors:** Marzena Szczygłowska, Anna Piekarska, Piotr Konieczka, Jacek Namieśnik

**Affiliations:** Department of Analytical Chemistry, Chemistry Faculty, Gdansk University of Technology, 11/12 Narutowicza Street, Gdansk 80-233, Poland; E-Mails: anna.m.piekarska@o2.pl (A.P.); piotr.konieczka@pg.gda.pl (P.K.); chemanal@pg.gda.pl (J.N.)

**Keywords:** brassica, heavy metals, phytoremediation, biofumigation

## Abstract

In recent decades, serious contamination of soils by heavy metals has been reported. It is therefore a matter of urgency to develop a new and efficient technology for removing contaminants from soil. Another aspect to this problem is that environmental pollution decreases the biological quality of soil, which is why pesticides and fertilizers are being used in ever-larger quantities. The environmentally friendly solutions to these problems are phytoremediation, which is a technology that cleanses the soil of heavy metals, and biofumigation, a process that helps to protect crops using natural plant compounds. So far, these methods have only been used separately; however, research on a technology that combines them both using white cabbage has been carried out.

## 1. Introduction

Increasing awareness of the hazards caused by environmental pollution has led to the search in many countries for methods, not only of recultivating land, but also preventing the contamination of the environment and food in the first place [1]. The following play a major part in environmental degradation:

- the dynamic growth of industry and transport;
- the non-rational use of pesticides in agriculture, of communal and industrial wastes, and of wastes for de-acidifying soils;
- human causes.

These give rise to deleterious changes in the natural landscape, pollute ground and surface waters, and reduce soil fertility [1]. Elevated levels of heavy metals in soil and plants have been measured in arable land, and this poses a threat to humans and animals. Hence, it is crucial to develop effective yet environmentally safe technologies for soil remediation. Interest is growing in methods of cultivation that do not make use of harmful chemicals. Many traditional technologies are extremely costly and time-consuming; other methods for cleaning up the environment require the use of other chemicals that may not always be benign with respect to the various compartments. An alternative to conventional technologies is phytoremediation, in which specially selected plants with a particular high affinity for heavy metals are used to restore degraded soils [2–4].

Environmental pollution seriously impairs soil quality. To maintain high crop yields, therefore, more and more pesticides are being used [5]; the vicious circle is thus closed as these simply compound the existing pollution. That is why alternative, environmentally friendly methods of protecting crops are being sought. One such method is biofumigation, which is based on the use of natural plant compounds to combat pests.

In this paper a review about about phytoremediation and biofumigation process with *Brassica* plants is presented; however, the innovative aspect of the present research lies in the possibility of combining these two processes using white cabbage.

## 2. Sources of Emission and Pathways of Environmental Contamination by Heavy Metals and Phytoremediation

Pollution of the natural environment by heavy metals may be a natural process due to the weathering of rocks or volcanic eruptions, but it can also be man-made [1,6]. Anthropogenic pollution by heavy metals is generally caused by some form of industry, transport, municipal waste management, landfill and the use of fertilisers. Contaminants can spread in the environment via the air, into which dust and gases are emitted, and through water and soil, onto which particles of contaminants are deposited from the air, or they are carried there by surface runoff and then percolate into the soil. Plants and animals can also convey harmful substances through the environment. The ultimate recipients are humans, because they breathe the air, drink the water and consume animal and vegetable foodstuffs. The threat of heavy metals to human and animal health is connected to their long-term persistence in the environment. For example, Pb, one of the more persistent metals, was estimated to have a soil retention time of about 150–5000 years and was reported to maintain high concentration for as long as 150 years after sludge application to soil. Also, the average biological halflife of Cd has been estimated as remaining about 10–18 years in the human body [7].

Heavy metals are major factors of soil pollution because:

- the contamination is frequently heterogeneous at the macroscale and microscale;
- the metals cannot be degraded biologically, but only transformed from one oxidation state or organic complex to another;
- the variability of metal forms and the soil matrix influence the environmental risk assessment and the soil treatment feasibility [8].

The soil-metal interactions depend on the specific metal form and soil characteristics such as particle size, cation exchange capacity, pH, soil mineralogy and organic content [1,8].

Metal concentrations in cultivated soil are lower than those recorded at industrial sites, but often high enough to generate a risk for environmental and human health. “In Germany about 10,000 ha of agricultural land would have to be taken out of food production because of heavy metal contamination exceeding these thresholds. The situation in Europe and in the USA probably *ca*. 100,000 ha of land are contaminated by heavy metals [9]”.

In view of this, comprehensive action is being taken to remediate contaminated land. Traditional remediation technologies with a chemical, physical or biological basis aim to:

- reduce an existing or potential environmental hazard;
- lower the potential threat from an unacceptable level to so-called ‘safe levels’.

However, even though these technologies are quite efficient in remediating contaminated land, they are too expensive and labor-intensive. An alternative technology, competitive with existing conventional methods, is phytoremediation. This makes use of the physical, chemical and biological processes that plants employ to absorb, accumulate and detoxify contaminants [2–4].

Plants have a natural ability to take up metals. Apart from cadmium, lead and mercury, which are always toxic at any level of the trophic pyramid, the heavy metals also include essential trace elements such as copper and zinc. In large amounts, however, both Cu and Zn are harmful to plants, animals and people [1]. Their phytotoxicity may be due to changes in physiological processes at the cellular and molecular level as a result of enzyme deactivation or the blocking of functional groups of metabolically important molecules. Quite often, metal poisoning leads to the elevated production of reactive oxygen species (ROS), which can damage macromolecular compounds in cells: proteins, lipids and nucleic acids [10]. Plants’ ability to accumulate heavy metals may cause problems to human health when contamination of food crops is too high. On the other hand, this ability forms the basis for phytoremediation.

Phytoremediation makes use of the ability of green plants to accumulate or degrade contaminants [11]. Phytoremediation can be carried out in a number of ways. In the process known as phytostabilization, plants convert contaminants to less assimilable forms, as a result of which the pollutants are not transported to the upper parts of the plants but remain locked in the rhizosphere. In phytodegradation, contaminants are decomposed within the plant following their uptake by the root system or outside the plant under the influence of plant enzymes secreted into the environment. Plants can also transform contaminants to usually less toxic, volatile forms, a process known as phytovolatalization. In phytostimulation, contaminants decompose in the presence of the micro-organisms present in the rhizosphere. Finally, there is phytoextraction, in which plants accumulate heavy metals in their above-ground organs [12–16].

Phytoremediation is regarded as the cheapest and environmentally most friendly technology for cleaning up soil. The most widespread and most profitable technique is phytoextraction, used mainly for removing heavy metals and radioactive elements from the soil [1]. Initially, the scope of phytoremediation was limited, principally because of the low bioavailability of heavy metals and the low biomasses of plants. Moreover, the management of the plant matter obtained after phytoremediation was troublesome [1,17,18]. Table 1 supplies information on the main problems and advantages that crop up in phytoremediation. There are many ways of improving the efficiency of this process, however. To enhance the accumulative potential of plants chelates can be used: these compounds substantially intensify the uptake and translocation of metals in plants in that they release metals from the soil and form soluble complexes with them, which are then transported by the xylem and deposited in the leaves. Uptake efficiency depends on the metals’ affinity for the chelate. The mobility of heavy metals in the soil can also be manipulated by altering its pH: a higher pH > 6.5 significantly reduces the quantity of readily soluble forms of metals in the soil and limits their uptake and accumulation by plants. By contrast, plants in an acidic environment can take up large amounts of these metals, even from soils that are only moderately polluted [1]. The success of phytoremediation depends mainly on the choice of plant, which must obviously possess the ability to accumulate large amounts of heavy metals (hyperaccumulation). These plants also have to satisfy other criteria:

➢ the concentration of heavy metals in the shoots should be 50–100 times greater than in ‘normal’ plants [19];
➢ the bioaccumulation coefficient (the ratio of the concentration of a toxic substance in the tissues of an organism to its concentration in the living environment of that organism) must have a value greater than 1 [20];
➢ metal concentrations in the shoots should be higher than in the roots [19];
➢ fast growth and high accumulating biomass [6];
➢ easily grown as an agricultural crop and fully harvestable [6].

Over 400 plant species have been identified as natural metal hyperaccumulators representing about 0.2% of all angiosperms. Unfortunately, most of these plants are characterized by slow growth and limited biomass production. Because of these limitations such plants cannot be used to remove certain heavy metals from soil. For instance, Pb phytoremediation technology can only be feasible if systems can be developed to employ high biomass plants, which are capable of accumulating more than 1% Pb in shoots and produce more than 20 t of biomass ha^−1^ yr^−1^ [20]. Based on the literature from 1995 until 2009, it can be stated that the most frequently cited species in phytoremediation studies was *Brassica juncea (L.)* Czern. (148 citations), followed by *Helianthus annuus L*. (57), *Brassica napus L.* and *Zea mays L*. (both 39 citations). The greater interest in *Brassicaceae* derives from the fact that research on these species started earlier, together with the interesting concentrations they provide, especially for *Brassica juncea* (L.) Czern [21]. Among the plants of the *Brassica* species, the *Brassica juneca* deserve special attention because its relevance to the process of phytoexctration of heavy metals from soil was confirmed in many experiments. It has been found that *B. juncea* exhibits a high capacity to accumulate Cd- mainly in the shoots, where Cd level was recorded at level of 1450 μg Cd/g dry wt. This is three times more than reported in *Brassica napus* (555 μg/g dry wt) [17]. In addition, this plant exhibit a high removal efficiency of other metals such as Pb (28% reduction) and Se (reduced between 13–48%) [3]. In addition, this plant is more effective at removing Zn from soil than *Thlaspi caerulescens*, a known hyperaccumulator of zinc. This is due to the fact, that *B. juneca* produces ten-times more biomass than *T. Cearullescens* [22]. However *Brassica juneca* needs to be harvested shortly after the plant becomes mature, which causes problems of disposal of obtained biomass. When these plants are dried, they easily crumble and flake off, greatly reducing the yield obtained, and the rest of the plant residues are a source of secondary emissions of toxic substances. Quite a large biomass and the lack of difficulties after harvesting are advantages described for different types of cabbage. In the case of Chinese cabbage, the high cumulative capacity of lead was observed within the limits of 5010 to 4,620 mg/kg dry wt. During testing capacity of phyoextraction of Zn, Cu and Pb for three *Brassica* crop species: *B. oleracea* L., *B. carinata* A. Br. and *B. juneca* (L.) Czern., the highest concentration of Zn (381 mg/kg dry wt.) and Cu (8,34 mg/kg dry wt.) were recorded in the shoots of *B. oleracea* L. The Pb concentrations of all *Brassica* species were more or less constant over the tested range of soil Pb concentrations, with lower values than the other metals. The low bioaccumulation of lead is due to its extreme insolubility and not generally being available for plant uptake in the normal range of soil pH [1,22].

The high potential of plants from the *Brassicaceae* family, which was presented above, for bioaccumulation of heavy metals along with management of plant matter after phytoremediation process, means that phytoremediation could become one of the most important technologies for cleaning the components from the environment. In recent years, interest in natural methods of plant protection against various pests has grown. Plants of the *Brassicaceae* family show great potential for use in biofumigaction.

## 3. Biofumigation as an Alternative Method of Crop Protection

Because the plants used for phytoremediation now contain high levels of contaminants in their tissues, they have become harmful waste that need to be appropriately disposed of. For a long time, the only way of doing this was incineration. Nowadays, however, this biomass is increasingly used to produce heat and electricity. In the case of brassicas cultivated on contaminated land, the plant matter can be disposed of in another way. Published research results indicate that the accumulation of heavy metals in cruciferous plants can stimulate the synthesis of glucosinolates (GLS), which are organic compounds containing sulfur. The products of their enzymatic degradation, mainly isothiocyanates, exhibit biocidal properties, which are used in biofumigation. The point of this process is that the volatile compounds with antibiological properties naturally occurring in brassica plants (*Brassicaceae*) are used to combat parasites, bacteria and fungi attacking crops. The concept of biofumigation is usually applied to these plants, because they contain considerable quantities of GLS. Furthermore, certain plant species of the *Brassica* family contain different levels of GLS with diverse compositions, so their hydrolysis products also have different biocidal activities [23].

**Figure 1:**
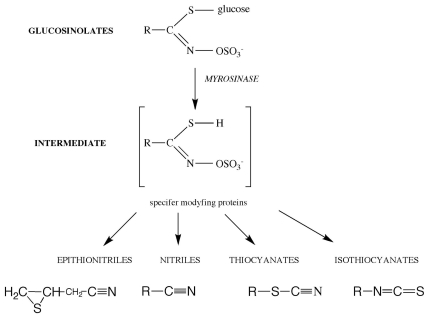
The products of the enzymatic hydrolysis of glucosinolates [24,25].

GLS themselves have no biocidal activity: it is their degradation products that do. GLS are hydrolyzed in a process catalyzed by the enzyme myrosinase (β-thioglucosidase, EC 3.2.3.1). In the plant cell, GLS are found in the vacuoles, whereas myrosinase is located separately in so-called myrosin cells. As a result of an attack by pests or mechanical damage, the cell structure is disrupted: GLS come into contact with the enzyme, and hydrolysis commences. The intermediate product of the reaction is thiohydroximate O-sulfonate, which, depending on the pH of the reaction medium, the presence of metal ions and additional protein factors, can be converted to isothiocyanates (ITC), thiocyanates, epithionitriles or nitriles (Figure 1) [24,25].

From the point of view of biofumigation, the most useful process is the degradation of GLS leading to the formation of isothiocyanates. These compounds display most powerful biocidal properties with a wide spectrum of activity: they attack fungi [26–31], herbivores [32–34] and bacteria [35–39] that cause leaves to darken or turn yellow, impair nutrient distribution in the plant, and cause fruit, stems and roots to rot. The activity of these pests leads to serious losses in agriculture and in crop storage, and reduces crop yields.

The structure of isothiocyanates is responsible for their efficacy: the more volatile the compound, the greater its antibiological activity due to better distribution. The type of microorganisms being combated, and even the particular phase of their growth, is also important [40,41]. The biocidal activity of isothiocyanates is comparable with the efficacy of synthetic pesticides, like methyl bromide, and some antibiotics (gentamycin) [33,39]. However, some synthetic pesticides, e.g., methyl bromide, have been withdrawn because they are harmful to human health and to the environment as they accumulate in organisms at different levels of the trophic pyramid. That is why the possibility of replacing pesticides with natural compounds with antibiological properties (i.a. isothiocyanates) seems so attractive and environmentally beneficial.

In addition, the compounds released into the soil during biofumigation can be a source of easily assimilable elements like carbon, nitrogen or sulfur.

To date, GLSs have been introduced to the soil mainly in two ways: by spreading dried and powdered parts of brassicas on fields [42,43] or by plowing them into the ground, treating the brassicas as a natural manure [32,44,45].

In one experiment selected *Brassica* crops, including canola, rapeseed, radish, turnip, yellow mustard and Indian mustard, were evaluated for control of various soilborne potato pathogens. All crops were seeded with a grain drill after disking, grown for 2–3 months and then plowed into the soil as a green manure. The powdery scab, main problem of potato fields, was reduced by Indian mustard, rapeseed and canola by 15–40%. Moreover canola and rapeseed reduced black scurf by 70–80% relative to a standard oats rotation. What is more, in *in vitro* assays, volatiles released from chopped leaf material of mentioned Brassica crops inhibited growth of a variety of soilborne pathogens of potato, including *Rhizoctonia solani, Phytophthora erythroseptica*, *Pythium ultimum, Sclerotinia sclerotiorum,* and *Fusarium sambucinam,* with Indian mustard resulting in nearly complete inhibition (80–100%) [32].

Antimicrobial activity of green manures of selected brassicas was also evaluated in an experiment with soil containing encysted eggs of *Globodera pallida*, a major pest of potatoes. The most effective was *Brassica juncea,* containing high concentrations of 2-propenylglucosinolate, and *Brassica rapa* (with 3-butenylglucosinolate as main GLS) causing over 91–95% mortality of encysted eggs of *G. pallida* [33].

The goal of another experiment was to determine the usefulness of dried leaves of savoy cabbage, red cabbage, horse radish and fringed cabbage in protection of cucumber against damping-off caused by fungi *Rhizoctonia solani* and *Fusarium culmorum.* The biggest fungistatic effect was observed in the case of horse radish, resulting in 100% inhibition of growth of *R. solani*. What is more, fungus *F. culmorum* was less sensitive for ITC activity than *R. solani* [45].

Also the *R. solani* growth inhibition by the Brassica species: *Diplotaxis tenuifolia* and *Brassica nigra* was determined in a next laboratory experiment. The addition of brassicas green manure to soil at 5% concentration suppressed the saprophytic growth of *R. solani* for about 82–87% comparing to control (no addition), after one month of trial duration [46].

These results indicate that Brassica crops have potential for use as green manures for the control of multiple soilborne disease problems. It is reasonable to search for new technologies to introduce the biofumigation process to agricultural practice.

## 4. Possibilty of Joining The Phytoremediation and Biofumigation Processes

**Figure 2:**
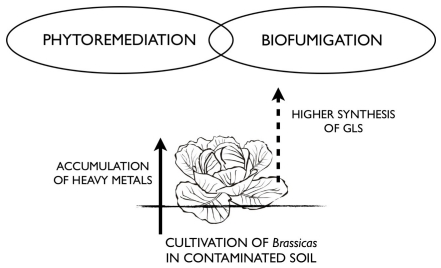
Diagram showing the combination of phytoremediation with biofumigation.

The presented review shows that Brassica plants are suitable as effective accumulators of heavy metals. Moreover these plants have antimicrobiological properties that can be used of as biofumigants for crop protection. Phytoremediation and biofumigation are usually carried out separately, which increases costs. Due to described features, the fundamental idea of joining these processes was conceived, using white cabbage (*Brassica oleracea* var. *capitata*), one the most popular European brassica plants. It is herein investigated whether cabbage used in phytoremediation might be suitable material for producing a biopreparate for plant biofumigation (Figure 2).

White cabbage was chosen because of the following benefits:

- grows quickly to produce a large biomass;
- is tolerant to environmental contaminants;
- contains metal-binding compounds such as glutathion, phytochelatin and metallothionein proteins;
- has an extensive root system;
- is an undemanding crop plant;
- is easy to harvest;
- has a large biomass enclosed in a small, compact spherical head, where the accumulated contaminants are safely stored until disposal. Even if a cabbage withers, it retains its structure and the dead leaves do not crumble, so there is no danger of the accumulated heavy metals returning to the environment;
- contains large amounts of glucosinolate sinigrin [47], the hydrolysis product of which is allyl isothiocyanate, a very effective biocide important in the biofumigation process.

More important still, the heavy metals taken up during phytoremediation by brassicas might intensify the synthesis of bioactive compounds, including GLS. The influence of metal accumulation on GLS levels and patterns, implies both direct and indirect metal-induced modification of GLS metabolism. Studies concerning the interactions of excess heavy metals with GLS plant content indicate their link to sulfur metabolism. However it is still not possible to provide a general balance of the influences of heavy metals on sulfur metabolism and the functioning of the sulfur pools in plants [48]. Metals such as Cd, Zn, Cu have been reported to induce the absorption of sulfate to sustain greater sulfur demand during the biosynthesis of GLS [49]. In Cd-tolerant *Thlaspi praecox* GLS level was increased by Cd exposure, mainly due to enhanced level of aromatic GLS, sinalbin. On the other hand a shift from alkenyl GLS to indolyl GLS was observed in Cd-treated sensitive *T. arvense* [50]. Zn can induce an increase in total GLS concentration in roots and decrease in shoots of *T. caerulescens*, however with reduced indolyl GLS levels both in roots and shoots [51]. Yet in *Brassica rapa* GLS level was decreased by Zn addition [52]. In contrast, Ni has no influence on GLS amounts in *Streptanthus polygaloides* [53]. Apparently the relationship between glucosinolates and metal accumulation is complex and involves metal, species and organ specific responses.

Not without significance is the fact that the alternating cultivation of cabbage and other vegetables is a long-standing practice for improving soil quality, so its use as a biofumigant should be readily accepted by farmers. The properties described above suggest that after cabbage has been used in phytoremediation, its use in the production of a biopreparate for crop protection by biofumigation is entirely justified. The large amount of water in a cabbage head should make it fairly easy to produce such a preparate, following the removal of the accumulated contaminants, which are probably bound to the solid parts rather than dissolved in the juice. These features appear to predispose cabbage for the environmentally friendly cleanup of soil in combination with a natural means of protecting plants.

## 5. Conclusions

The ability of brassicas to bioaccumulate heavy metals can be used to reduce the level of contaminants in the soil (phytoremediation), and thus to clean up and prepare soils for cultivation. On the other hand, the antimicrobial activity of these plants means that thay can be used as a natural fungicide and bacteriostatic and in this way improve crop yield and soil quality.

## Figures and Tables

**Table 1:** The main advantages and limitation of phytoremediation technology [4,14–16].

Advantages	Limitations
Applicable to both inorganic and organic contaminants.	Not accessing elements below the root depth.
It can be applied *in situ*.	Management of plant matter after phytoremediation.
Reduces the amount of waste going to landfills.	Low bioma.
Does not require expensive equipment or highly specialized personnel.	The bioavailability of the pollutants.
Phytoremediation is cheaper than conventional remediation methods.	Restricted to sites with low contaminant concentration.
Easy to implement and maintain. Plants are a cheap and renewable resource, easily available.	Introduction of inappropriate or invasive plant species should be avoided (non-native species may affect biodiversity).
Environmentally friendly, socially accepted.	High concentrations of hazardous materials can be toxic to plans.
Less noisy than other remediation methods.	Possibility for contaminants to enter food chain through animal and plant consumption.
